# A unique midgut-associated bacterial community hosted by the cave beetle *Cansiliella servadeii* (Coleoptera: Leptodirini) reveals parallel phylogenetic divergences from universal gut-specific ancestors

**DOI:** 10.1186/1471-2180-13-129

**Published:** 2013-06-10

**Authors:** Maurizio G Paoletti, Luca Mazzon, Isabel Martinez-Sañudo, Mauro Simonato, Mattia Beggio, Angelo Leandro Dreon, Alberto Pamio, Mauro Brilli, Luca Dorigo, Annette Summers Engel, Alessandra Tondello, Barbara Baldan, Giuseppe Concheri, Andrea Squartini

**Affiliations:** 1 Dipartimento di Biologia, Università di Padova, via U. Bassi 58/B, Padova, 35131, Italy; 2 Dipartimento di Agronomia Animali Alimenti Risorse Naturali e Ambiente, Università di Padova - Agripolis, Viale dell’Università, Padova, Legnaro, 16 - 35020, , Italy; 3 Istituto di Geologia Ambientale e Geoingegneria, Dipartimento di Scienze della Terra, Università La Sapienza di Roma, Rome, 00185, Italy; 4 Natural History Museum, Via Marangoni 39, Udine, 33100, Italy; 5 Department of Earth and Planetary Sciences, University of Tennessee, Knoxville, TN, 37996,, USA

**Keywords:** *Cansiliella servadeii*, Gut bacteria, Animal-bacteria coevolution, Cave, Moonmilk, Food web

## Abstract

**Background:**

*Cansiliella servadeii* (Coleoptera) is an endemic troglobite living in deep carbonate caves in North-Eastern Italy. The beetle constantly moves and browses in its preferred habitat (consisting in flowing water and moonmilk, a soft speleothem colonized by microorganisms) self-preens to convey material from elytra, legs, and antennae towards the mouth. We investigated its inner and outer microbiota using microscopy and DNA-based approaches.

**Results:**

Abundant microbial cell masses were observed on the external appendages. *Cansiliella*’s midgut is fully colonized by live microbes and culture-independent analyses yielded nearly 30 different 16S phylotypes that have no overlap with the community composition of the moonmilk. Many of the lineages, dominated by Gram positive groups, share very low similarity to database sequences. However for most cases, notwithstanding their very limited relatedness with existing records, phylotypes could be assigned to bacterial clades that had been retrieved from insect or other animals’ digestive traits.

**Conclusions:**

Results suggest a history of remote separation from a common ancestor that harboured a set of gut-specific bacteria whose functions are supposedly critical for host physiology. The phylogenetic and coevolutionary implications of the parallel occurrences of these prokaryotic guilds appear to apply throughout a broad spectrum of animal diversity. Their persistence and conservation underlies a possibly critical role of precise bacterial assemblages in animal-bacteria interactions.

## Background

The associations between microorganisms and insects are widespread in nature [1,2]. Relationships between obligate symbioses and instances of co-evolution have been reported for mealybugs [3], whiteflies [4], weevils [5], tsetse flies [6], cockroaches and termites [7], aphids [8], planthoppers [9], carpenter ants [10]. In previous work of ours we have examined a number of symbiotic occurrences within dipterans, describing the novel species ‘*Candidatus* Erwinia dacicola’ dwelling in the oesophageal bulb of the olive fly [11,12] and the novel genus *Stammerula,*[13]; for which we highlighted evidences of joint evolution with the insects [14,15].

Hosting bacteria can result in different benefits for insects, among which a specific nutritional complementation is critical for those living on a markedly imbalanced diet, e.g. aphids [16] or ants. In the latter example trophic metabolism has been recognized as a major contributor of evolutionary shifts [17], as in the case of the *Tetraponera* ants [18]. In these ants the onset of herbivory has been postulated to be the result of the link with internal bacteria. Further examples include other hymenoptera whereby members of the characteristic bacterial microbiota of the honey bee *Apis mellifera* were absent from most species outside of the corbiculate bees, and a specific co-evolution between these hymenoptera and a defined bacterial guild was postulated to explain such association [19]. All of these relationships have also been hypothesized to involve oxidative recycling of nitrogen-rich metabolic waste and are encaged in specialized hindgut- or midgut-derived pouches. Stinkbugs host *Burkholderia* in their midgut crypts [20,21], while the medicinal leech carries *Aeromonas* and a member of the Rickenellaceae in its intestinal assemblage [22,23].

**Figure 1:**
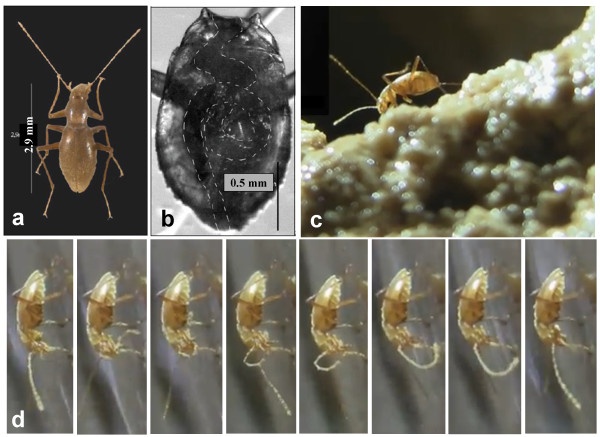
***Cansiliella servadeii *and its habitat. a**) Top view of the adult insect. **b**) detail of the abdomen with indication of the gut position and coiling; **c**) insect browsing on moonmilk in Grotta della Foos cave floor. **d**) sequence showing *C.servadeii* on location, preening its left antenna and passing it through mouthparts.

For invertebrates that permanently live in secluded habitats with little exchange with the external biota, such as cave environments, the importance of microsymbionts can be particularly critical for host adaptation and survival. Some cave-dwelling animals owe their life to symbioses with chemolithoautotrophic bacteria [24,25]. We previously described a novel genus and two species of a troglobitic beetle, *Cansiliella tonielloi*[26,27] and *Cansiliella servadeii* (Figure 1a) [28], which are endemic of few karst caves in Northern Italy. The latter has been the object of more detailed studies [29,30], where we further described the physico-chemical features of its environment.

The beetles live in a hygropetric habitat in the presence of a peculiar, soft speleothem called moonmilk, which consists of carbonate minerals that are constantly covered by a thin layer of running water [31]. This habitat type is common in air-filled caves, and is typified by dripwaters or sheetflow that bring allochthonous, surface-derived organic matter [32]. Hydrological isolation for some cave hygropetric habitats may restrict the influx of organic matter, and this can lead to nutritional limitations for troglobites and troglophiles over extended periods of time and be a major driver for evolutionary adaptation for troglobites [32].

Moonmilk usually carries high amounts of microbial biomass [33-38]. In the Grotta della Foos, one of the cave systems being studied, the wet moonmilk contains ~10^8^ microbial cells/ml and ~10^4^ meiofaunal cells/m^2^ and its bacterial community characterization is described in a parallel study of ours [39]. The insect spends most of its time browsing the moonmilk surface and frequently self-preening. Videos of live *C. servadeii* in Grotta della Foos (http://www.youtube.com/watch?v=iXF5pDrF2J0) were taken, and its activities and behaviour were recorded. The mouthparts are consistent with reported models of adaptation for browsing/filtering organic particles in semi-aquatic environments [40], and differ markedly from those of the majority of other troglobitic Leptodirini [32,41-43]. The mouthparts have modified hoe-shaped mandibles and spoon-like galeae covered by dense setae spaced 1–1.5 μm. This distance could effectively rake particles of compatible size, such as bacteria [29,30].

Our previous stable isotope investigations [30] demonstrated that *C. servadeii* derives its nutritional requirements from the moonmilk and from dissolved organic matter in the percolating waters. To our knowledge, there are no molecular studies of the gut microbiota of cave invertebrates. The current project aimed at characterizing the feeding behaviour of *C. servadeii* from Grotta della Foos and the nature of its gut microbiota. The results provided insights pointing towards the existence of a universal guild of bacteria which appears to be common to many animal digestive systems and that suggests to have shared ancestors established prior to their hosts evolution.

## Methods

### Sampling site, specimen observation and collection

The Grotta della Foos cave system formed within Monte Ciaurlec located in north-eastern Italy, and is underlain by Cretaceous and Triassic limestone units [44] The cave contains over 2600 m of passages. Ten sampling locations within the cave were used for the investigations of behaviour and insect collection. the sites covered altogether 13.3, square meters, which is the whole area which *Cansiliella* is regularly found in Grotta de la Foos cave. The density monitored varied from 1.4 to 1.8 specimens per square meter. Examined specimen were all adults and included both sexes. Live *C. servadeii* were collected in sterile falcon tubes and transported to the laboratory.

### Microscopy, insect dissection, and gut content evaluation

Insects external teguments were stained with DAPI (5 μg/ml) and observed in visible light and in epifluorescence using a Leica DM4000 inverted microscope equipped with a DFC300 FX camera. Images were acquired by using the LAS software.

Insects were dissected to remove the midgut to analyze the intestinal microflora. Before dissection, specimens were stunned by keeping vials at 4°C for 20 min. To extract the midgut, the insect’s abdomen was opened under a stereomicroscope (Figure 1b) in a laminar flow hood using sterile equipment and sterile water. The midgut was transferred in a sterile Eppendorf tube and used for both bacterial culturability tests and bacterial DNA extraction and amplification, and was stored at −20°C until extraction.

A segment of each midgut was observed under microscopy after staining with the LIVE/DEAD® BacLight Bacterial Viability Kit (Molecular Probes, California, USA). Slides were also prepared for Gram staining and morphological characterization, which was performed under an Olympus BX60 microscope.

### Bacterial cultivation

In order to examine external bacteria adhering to the insect exoskeletal tegument, live specimens collected with cave water in falcon tubes were handled with sterile forceps and gently touched over the surface of Plate Count Agar (PCA) (Oxoid) plates.

The possible culturability of the microorganisms hosted in the insect midgut was verified by plating aliquots of resuspended, dissected gut material extracted onto PCA plates.

All plates were incubated in the dark at 20°C for up to 10 days.

### DNA extraction, 16S rRNA gene amplification, cloning, and sequencing

DNA was extracted from the content of the midguts, as previously described [45]. PCR amplification targeting the 16S rRNA gene was carried out in 20 μl, 1x PCR GoTaqFlexi Buffer (Promega), 2.5 mM MgCl2, 0.1 mM dNTPs, 0.5 μM of each primer, 1 U of GoTaq Flexi DNA polymerase (Promega), and 1 μl of a 1:30 dilution of the DNA extraction. The universal bacterial 16S rRNA primers used were 63f and 1389r [46] to yield an expected amplicon of ~1300 bp. The cycling program consisted of a 95°C 2 min step followed by 35 cycles at 96°C for 30 s, 56°C for 30 s, 72°C for 90 s, and a final extension at 72°C for 10 min. PCR products were checked by 1.0% agarose gel stained with SYBR®Safe (Invitrogen) and purified with the ExoSAP-IT kit (Amersham Biosciences) before sequencing.

Amplicons (1300 bp) were cloned into JM109 competent cells using the P-GEM-T Easy vectors (Promega), following the manufacturer’s recommendations. Thirty clones from each of the three gut specimen samples were picked. Transformation was verified using PCR assays with the M13-T7 universal primers pair. The amplification products were sequenced by capillary electrophoretic sanger sequencing using M13 and T7 primers at the BMR Genomics service (Padova, Italy). Restriction enzyme (*Bsa*I, Euroclone) digestion patterns of the amplified 16S gene (ARDRA) were used as a parallel clone screening in addition to nucleotide sequencing.

### Sequence analysis

Sequence chromatograms were visually inspected and sequences were edited and aligned by using MEGA 4.0.2 (http://www.megasoftware.net/).

Chimeras were searched with the CHIMERA CHECK program of the Ribosomal Database Project II (http://rdp.cme.msu.edu).

A BLASTN GenBank analysis of all the sequences was done through the NCBI website (http://www.ncbi.nlm.nih.gov/) and closely related sequences from the databases were retrieved and added to the alignment. Phylogenetic relationships among newly retrieved gut microbiota sequences to close relatives were estimated using a maximum likelihood analysis (ML) with a GTR+I+G model.

The software package DOTUR [47] was used to assign sequences to operational taxonomic units (OTUs) for the bacterial identities found in the midgut of *C. servadeii*. This program assigns sequences to OTUs based on sequence data by using values that are less than the cut-off level, which were at the 97% and 95% identity thresholds. The Chao1 richness estimator [48] was also calculated using DOTUR. The richness estimates are reported for 3% difference between sequences.

16S rRNA gene sequences of clones from the guts of *C. servadeii* are accessible under numbers JQ308110 to JQ308155 and from JX463074 to JX463100.

The sequences from the culturable microbial community from the midgut and the external tegument are accessible under numbers JQ308156 to JQ308165.

## Results

### Observations of insect behaviour

Live activities were monitored for *C. servadeii* individuals within Grotta della Foos on six different expeditions (Figure 1). Consistent behavioural patterns could be defined from a continuous 24-hour period from eight specimens. The insect spends 44% of the time at a depth between 4 and 20 mm under the water that flows over the moonmilk speleothem. During this activity, the mouthparts and head are engaged in a prolonged browsing to rubbing motion (Figure 1c). Nearly half of the time was dedicated to self-preening of the head, legs, elytra and antennae; this behaviour is suggestive of a feeding activity as it moves organic particulates from the body towards the mouth. Typically, during preening, the insect passed the posterior legs over the elytra, then the middle legs brushed the posterior ones, the forelegs brushed the middle ones, each antenna, and then the forelegs passed between the mandibles and galeae. Antennae were combed for their entire length, as shown by the consecutive frames of the sequential series (Figure 1d), taken from footage available at http://www.youtube.com/watch?v=iXF5pDrF2J0. The observed aquatic and semi-aquatic movement actively displaced superficial sediment granules and disrupted moonmilk into trenches ~0.2 to 3 mm long.

**Figure 2:**
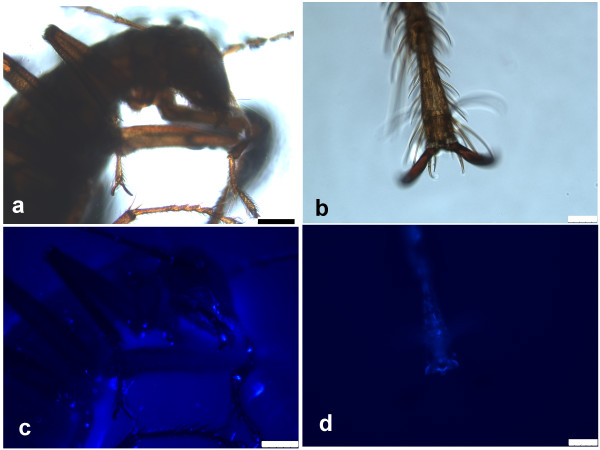
***Cansiliella servadeii *observation under epifluorescence stereomicroscope after staining with the DNA-specific DAPI fluorochrome. a**), **c**): head and torso view; **b**), **d**) detail of foreleg underside. **a**), **b**): white illumination; **c**), **d**): UV illumination. The presence of masses of bacteria staining with DAPI on the insect head, limbs, antennae and ventral side of body is visible. Scale bars: **a**), **c**): 250 μm; **b**), **d**): 50 μm.

In support of the hypothesis that the browsing and preening activities are related to feeding, possibly to acquire organic matter or cellular material from the wet moonmilk, the DAPI fluorescent stain shows that the hair-covered upper underside and interior legs of the insect body parts, that are continuously rubbed during preening, are covered by masses of bacteria-containing material (Figure 2). Crawling across the soft moonmilk, and passing the antennae tightly by the mouthparts, as shown by the sequence in Figure 1d, contributes to scooping up abundant organic material visible on the ventral segment of the body (Figure 2c).

### Presence and viability of midgut bacteria

**Figure 3:**
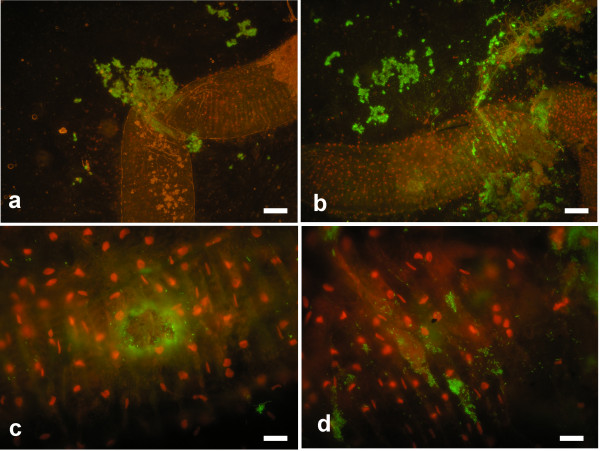
**BacLight staining of dissected *Cansiliella servadeii *midgut resuspended material.** Live bacterial cells stain in green while insect epithelial nuclei stain in red. In **a**) clumps of bacteria are seen flowing out from the rupture of the bent gut tract. In **b**) a different portion is shown and the abundant masses of extracted bacteria. In **c**) individual bacterial cells are released from the gut epithelium through a hole pierced with forceps. In **d**) a region of the gut from which several distinct bacterial cells can be seen along with others in more clustered formations. Scale bars: **a**),**b**): 350 μm,**c**),**d**): 50 μm*.*

We explored *C. servadeii* midgut (Figure 1b) by pulling it out gently from dissected specimens and staining it with the Bac/Light live-dead bacterial stain. The results shown in Figure 3, reveal that abundant alive (green-staining), prevailingly rod-shaped, bacterial cells fill the lumen of the gut. In the images, in which the nuclei of the insect epithelial layers stain in red, profuse live bacterial content is seen oozing out from the gut tube in correspondence of its ruptures. In Figure 3c a hole was pierced with forceps on the gut wall, through which a lump of bacterial cells are consequently pouring out. The data indicate that this cave beetle hosts live prokaryotes in its digestive tract. In order to investigate their identities we proceeded with both culture-dependent and independent approaches as follows.

### Culturable microbial community from the external tegument and midgut

Touching the external tegument of wet live specimens onto PCA plates resulted in colonies that belonged to four 16S phylotypes representing three lineages (Gammaproteobacteria, Actinobacteria, and Firmicutes) (Table 1).

**Table 1:** **Taxonomical assignment based on 16S rRNA gene sequencing of culturable isolates from the external exoskeleton of *Cansiliella servadeii *(non-surface sterilized specimens) or from its midgut content (surface-sterilized specimens)**

	**Taxonomy**	**Isolate, GenBank code**	**Top database similarities (%)**^**1**^	**Habitat of subject**^**2**^
Tegument	γ-*Proteobacteria*	InGrP, (JQ308165)	(100) *Pseudomonas* sp. EU182834	Soil
	Actinobacteria	InGrG, (JQ3081649)	(99.4) *Streptomyces* sp. JF292927	Endophyte in *Lobularia* sp*.*
	Actinobacteria	InGrA3, (JQ308163)	(99.4) *Rhodococcus*sp. HQ256783	Cloud water from mountain summit
	Firmicutes	InGrA1, (JQ308162)	(96.8) Unc.bacterium JF107304	Human skin, antecubital fossa
Midgut	γ-*Proteobacteria*	CP1a, (JQ308158)	(100) *Pseudomonas* sp. AB569967	Chitinolitic biota in rhizosphere soil
	γ-*Proteobacteria*	CP1b, CP2b, (JQ308159)	(100) *Pseudomonas* sp. AJ243602	*Lumbricus rubellus* gut (Annelida)
	Actinobacteria	CP2a, CP3aL, (JQ308160)	(100) *Streptomyces champavatii* HQ143637	Soil
	γ-*Proteobacteria*	CP3a, (JQ308161)	(100) Unc. *Pseudomonas* sp. JF500897	Rye grass rhizosphere
	Firmicutes	CP4.1, CP4.2, (JQ308156)	(99.4) Unc. Firmicutes EU005283	Inert surfaces immersed in marine water
	Firmicutes	CP4.3, (JQ308157)	(98.6) Unc.bacterium DQ860054	Anchovy intestinal microflora

^1^Description of GenBank subjects displaying the top-scoring BLAST alignment results of sequence similarity.

^2^Animal host or other environment in which the subject having homology with the present sequence s described in GenBank records.

From the extracted insect guts, there were sparse colonies that grew on PCA plates, and the most frequent morphological colony type resulted in isolate CP4.1. Sequences obtained from the external tegument, as well as from the culturable fraction of midgut bacteria, had high homology values (in most cases 99-100%) to bacterial taxa featuring multiple occurrencies in the nucleotide sequence database (for references consult the GenBank accession numbers given in the fourth column of Table 1), although there was no close affinity to sequences previously retrieved from insect guts.

### Culture-independent analysis of the midgut microbial community

**Figure 4:**
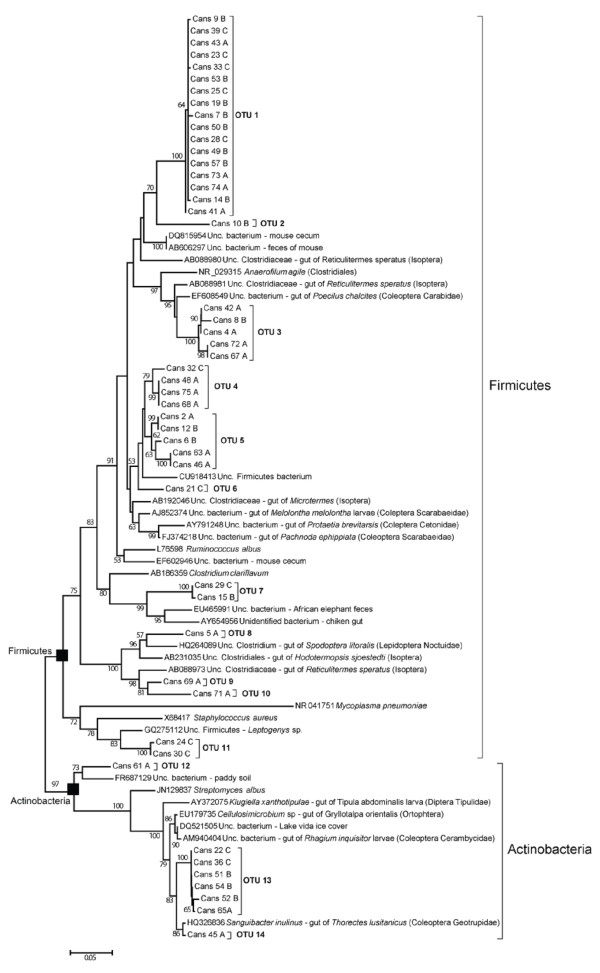
**Maximum likelihood tree of 16S rRNA gene clone sequences recovered of the midgut of *Cansiliella servadeii *affiliated with gram-positive bacteria.** The sequences of GenBank dataset showing the closest similarity levels have been added. The percentage of replicate trees in which the associated taxa clustered together in the bootstrap value shown next to the branches. Only values greater than 50 are indicated. All positions containing gaps and missing data were eliminated from the dataset (Complete deletion option).

**Figure 5:**
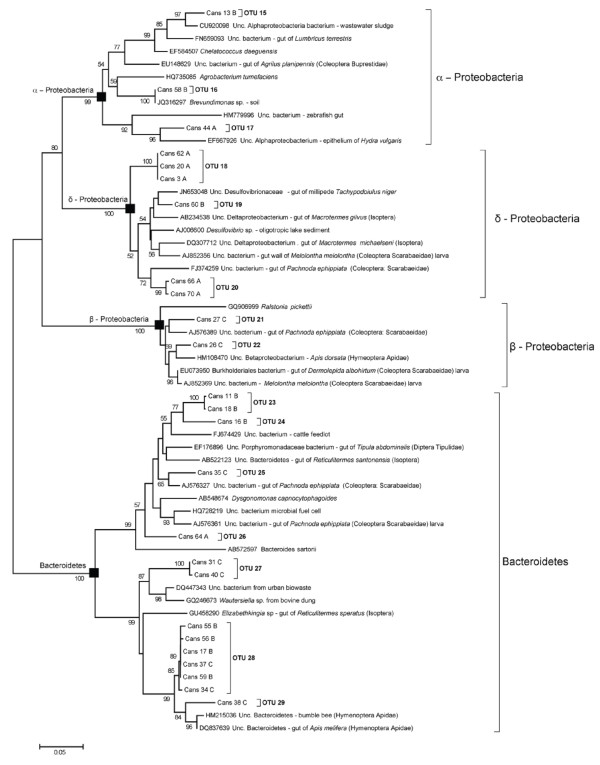
**Maximum likelihood tree of 16S rRNA gene clone sequences recovered of the midgut of *Cansiliella servadeii *affiliated with Proteobacteria and Bacteriodetes.** Sequences from GenBank dataset showing the closest similarity levels have been added. The percentages of replicate trees in which the associated taxa clustered together in the bootstrap test are shown next to the branches. Only values higher than 50 are indicated. All positions containing gaps and missing data were eliminated from the dataset (Complete deletion option).

Under the limitations posed by working with a rare endemic and protected species with minimum sampling allowed, we analyzed three specimens from which separate clone libraries of 16S rRNA gene amplicons were generated and 87 clones screened. Sequences from the three different guts are labeled with the suffixes A, B, and C, respectively, on Table 2. At this resolution level the number of Dotur-defined species was 29 and the Chao1 estimator [48] predicted a total number of species of 51,7. We also calculated the estimated coverage by applying the Good’s index [49] which, at species level, resulted 81.6 %. In order to check with an independent method whether the sampling size had been truly effective in yielding an adequate representation of the community, we compared the cluster analysis dendrogram obtained with the first 46 clones screened (Additional file 1: Material S1 and Additional file 2: Material S2) with those generated with the whole set of 87 (Figures 4 and 5), from whose comparison it can be observed that the community structure was already fully delineated from the first stepwise subset of randomly selected clones. Further, considering the phylum rank as a more functional assessment of population diversity we run rarefaction curves with OTUs defined at a phylum level similarity threshold (81%). The result obtained indicated a saturating curve and is shown in the supplementary Additional file 3: Figure S3.

**Table 2:** **Taxonomical assignment of cloned 16S rRNA amplicons from the midgut content of *Cansiliella servadeii***

**OTU #**	**Taxonomy**	**Clone and GenBank Code**	**Top database similarities (%)**^**1**^	**Habitat**^**2**^
1	Firmicutes	7B, (JQ308118)	(92.4) Unc^3^. bacterium AB606297	Mouse faeces
			(92.1) Unc. Clostridiaceae AB088980	*Reticulitermes speratus* gut (Isoptera: Termitidae)
		43A;14B; 9B; 33C, (JQ308112, JQ308119, JQ308111, JQ308113)	(92.6) Unc. bacterium AB606297	Mouse faeces
			(92.4) Unc. bacterium DQ815954	Mouse cecum
			(92.3) Unc. Clostridiaceae AB088980	*R. speratus* gut
		19B; 23C; 25C; 28C; 39C, 50B, 53B, 57B, 73A, 74A (JQ308115, JQ308116, JQ308110, JQ308114, JQ308117, JX463078, JX463086, JX463088, JX463089), JX463090	(92.9) Unc. bacterium AB606297	Mouse faeces
			(92.6) Unc. Clostridiaceae AB088980	*R. speratus* gut
		41A, (JQ308120)	(93.1) Unc. bacterium AB606297	Mouse faeces
			(92.9) Unc. bacterium DQ815954	Mouse cecum
			(92.8) Unc. Clostridiaceae AB088980	*R. speratus* gut
		49B (JX463074)	(92.9) Unc. bacterium AB606297	Mouse faeces
			(92.6) Unc. bacterium DQ815954	Mouse cecum
			(92.5) Unc. Clostridiaceae AB088980	*R. speratus* gut
2	Firmicutes	10B, (JQ308121)	(92.3) Unc. bacterium EF602946	Mouse cecum
3	Firmicutes	4A; 42A, (JQ308123, JQ308124)	(95.9) Unc. Clostridiales AB088981	*R. speratus* gut
			(94.4) Unc. bacterium GU451010	*Tipula abdominalis* gut (Diptera: Tipulidae)
		67A, 72A (JX463084, JX463085)	(94.8) Unc. Clostridiales AB088981	*R. speratus* gut
		8B, (JQ308122)	(95.5) Unc. bacteriumEF608549	*Poecilus chalcites* gut (Coleoptera: Carabidae)
4	Firmicutes	32C, (JQ308126)	(95.2) Unc. Clostridiaceae AB192046	*Microcerotermes* spp. gut (Isoptera: Termitidae)
		48A, 68A, 75A (JQ308127, JX463080, JX463091)	(95.7) Unc. bacterium AJ852374	*Melolontha melolontha* gut (Coleoptera: Scarabaeidae)
5	Firmicutes	21C, (JQ308125)	(94,5) Unc. bacterium FJ374218	*Pachnoda* spp*.* gut (Coleoptera: Scarabaeidae)
6	Firmicutes	2A;12B, (JQ308128, JQ308129)	(97.1) Unc. Clostridiaceae AB192046	*Microcerotermes* spp. gut (Isoptera: Termitidae)
		6B, (JQ308130)	(96.9) Unc. bacterium FJ374218	*Pachnoda* spp. larval gut (Coleoptera: Scarabaeidae)
		46A, 63A (JQ308131, JX463079)	(94.5) Unc. bacterium FJ374218	*Pachnoda* spp*.* gut (Coleoptera: Scarabaeidae)
7	Firmicutes	15B, (JQ308133)	(91.7) Unc. bacterium EU465991	African elephant faeces
			(90.5) Unc. bacterium AY654956	Chicken gut
		29C, (JQ308132)	(91.9) Unc. bacterium EU465991	African elephant faeces
			(90.7) Unc. bacterium AY654956	Chicken gut
8	Firmicutes	5A, (JQ308134)	(93.8) Unc. Clostridiales AB231035	*Hodotermopsis sjoestedti* gut (Isoptera: Termitidae)
9	Firmicutes	69A (JX463081)	(94.7) Unc. bacterium AB088973	*R. speratus* gut
10	Firmicutes	71A(JX463087)	(92.7) Unc. bacterium AB088973	*R. speratus* gut
11	Firmicutes	24C, 30C, (JQ308135, JQ308136)	(92.6) Unc. Firmicutes GQ275112	*Leptogenys* spp. gut (Hymenoptera: Formicidae)
12	Actinobacteria	61A (JX463076)	(93.2) Unc. Bacterium FR687129	Paddy soil
13	Actinobacteria	22C; 36C, 51B, 54B (JQ308137, JQ308138, JX463075, JX463083)	(97.2) Unc. bacterium DQ521505	Lake Vida ice cover
			(96.9) Unc. bacterium AM940404	*Rhagium inquisitor* gut (Coleoptera: Cerambycidae)
		52B (JX463077)	(96.7) Unc. bacterium DQ521505	Lake Vida, ice cover
			(96.5) Unc. bacterium AM940404	*Rhagium inquisitor* gut (Coleoptera: Cerambycidae)
		65A (JX463082)	(97) Unc. bacterium DQ521505	Lake Vida, ice cover
			(96.7) Unc. bacterium AM940404	*Rhagium inquisitor* gut (Coleoptera: Cerambycidae
14	Actinobacteria	45A, (JQ308139)	(99.5) *Sanguibacter inulinus* HQ326836	*Thorectes lusitanicus* gut (Coleoptera: Geotrupidae)
15	α-*Proteobacteria*	13B, (JQ308142)	(96.2) Unc. α-proteobacterium CU920098	Mesophilic anaerobic digester treating wastewater sludge
			(93.7) Unc. bacterium FN659093	*Lumbricus terrestris* gut
16	α-*Proteobacteria*	58B (JX463098)	(100) Brevundimonas sp.JQ316297	Soil
17	α-*Proteobacteria*	44A (JQ308143)	(92.5) Unc. bacterium EF667926	Epithelium *Hydra vulgaris*
			(88.2) Unc. bacterium HM779996	Adult zebrafish gut
			(87.9) Unc. bacterium EU148629	*Agrilus planipennis* gut (Coleoptera: Buprestidae)
18	δ-*Proteobacteria*	3A; 20A, 62A (JQ308144, JQ308145, JX463096)	(94.3) Unc. δ-proteobacterium DQ307712	*Macrotermes michaelseni*gut (Isoptera: Termitidae)
19	δ-*Proteobacteria*	60B (JX463100)	(96) Unc. Desulfovibrionaceae JN653048	Gut of millipede *Tachypodoiulus niger*
20	δ-*Proteobacteria*	66A, 70A (JX463092, JX463093)	(94.1) Unc. bacterium FJ374259	*P. ephippiata* gut (Coleoptera: Scarabaeidae)
21	β-*Proteobacteria*	27C, (JQ308141)	(95.2) Unc.bacterium AJ852369	*Melolontha melolontha* gut (Coleoptera: Scarabaeidae)
22	β-*Proteobacteria*	26C, (JQ308140)	(96.5) Burkholderiales bacterium EU073950	*Dermolepida albohirtum* gut (Coleoptera: Scarabaeidae)
23	Bacteroidetes	11B, (JQ308146)	(91.9) Unc. bacterium AJ576327	*Pachnoda ephippiata* gut (Coleoptera: Scarabaeidae)
		18B, (JQ308147)	(92.1) Unc. bacterium HQ728219	Microbial fuel cell
			(91.9) Unc. bacterium AJ576327	*P. ephippiata* gut (Coleoptera: Scarabaeidae)
24	Bacteroidetes	16B, (JQ308148)	(92.5) Unc. bacterium FJ674429	Cattle feedlot
			(91.9) Unc. Bacteroidetes AB522123	*R. santonensis* gut (Isoptera: Termitidae)
			(89.2) Unc. bacterium EF176896	*Tipula abdominalis* gut (Diptera: Tipulidae)
25	Bacteroidetes	35C, (JQ308149)	(96.2) Unc. bacterium AJ576327	*P. ephippiata* gut (Coleoptera: Scarabaeidae)
26	Bacteroidetes	64A (JX463097)	(94.2) Unc. bacterium HQ728219	Anode of a glucose-fed microbial fuel cell
			(93.7) Unc. bacterium AJ576361	*P. ephippiata* gut (Coleoptera: Scarabaeidae)
27	Bacteroidetes	31C, (JQ308150)	(93.1) Unc. bacterium DQ447343	Urban biowaste
			(89.3) *Elizabethkingia* sp. GU45829	*R. speratus* gut (Isoptera: Termitidae)
		40C, (JQ308151)	(92.8) Unc. bacterium DQ447343	Urban biowaste
			(89.2) Unc. Bacteroidetes HM215036	Bumble bee gut (Hymenoptera: Apidae)
28	Bacteroidetes	17B; 37C; 34C, 59B (JQ308154, JQ308155, JQ308153, JX463099)	(94.9) Unc. Bacteroidetes DQ837639	*Apis mellifera* gut (Hymenoptera: Apidae)
		55B (JX463095)	(94.6) Unc. Bacteroidetes DQ837639	*Apis mellifera* gut (Hymenoptera: Apidae)
		56B (JX463094)	(94.8) Unc. Bacteroidetes DQ837639	*Apis mellifera* gut (Hymenoptera: Apidae)
29	Bacteroidetes	38C, (JQ308152)	(94.3) Unc. Bacteroidetes DQ837639	*Apis mellifera* gut (Hymenoptera: Apidae)

^1^Description of GenBank subjects displaying the top-scoring BLAST alignment results of sequence similarity.

^2^Animal host or other environment in which the subject having homology with the present sequence is described in GenBank records.

^3^Unc. = ‘Uncultured’.

OTUs are defined at 97% similarity threshold. Clones ID are followed by letters A,B or C to identify the three insect guts specimens.

Phylogenetic analyses revealed the presence of six distinct major phylogenetic groups from the sequenced clones.

The sequences showed a range of homology values with the GenBank database records that for most cases was remarkably low (Table 2).

Considering the totality of the 87 clones, the Firmicutes phylum represented 58,6% of all retrieved sequences, and over 60% of the clones showed homologies as low as 92-94% with existing database subjects. Bacteroidetes represented 16.1% of the sequences, with homologies 89-94% to GenBank entries. Only few clones of the Actinobacteria (whose phylum represented 11.5% of the retrieved sequences) displayed similarity values qualifying for species level relatedness (≥97%) with described records.

The remainder of the clones were affiliated with the Deltaproteobacteria (8.0%) and with the Alpha- and Betaproteobacteria, classes (<5% each). Although culturable strains affiliated to the Gammaproteobacteria were obtained from the gut (Table 1), no clone sequences affiliated with this class were retrieved, presumably due to their rarity within the total community.

The taxonomical groups resulted homogeneously distributed through the samples analyzed. There was no statistical difference in the distribution of the phylogenetic groups of Firmicutes, Actinobacteria, Proteobacteria and Bacteroidetes from the different midgut samples (Fisher’s exact test, *P* = 0.22). All guts had an outstanding majority of OTUs belonging to the Firmicutes.

Although the BLAST analysis gave similarities that in most cases were below the species and even genus limit (respectively for the 89.04% and 63% of the samples), nevertheless the best matches of a vast majority of clones corresponded to bacteria occurring in different insects gut, including ants, termites, and beetles (Table 2). It is worth adding that more than 80% of these hosts spend at least part of their life cycle in the soil, and ~46% of them belong to the Coleoptera order (Carabidae, Scarabaeidae and Geotrupidae). Another key finding is the fact that groups of taxonomically distinct clones from *C. servadeii* have their respective GenBank matches in sequences that were found also in the same insect host species. For example, three non-identical Clostridiales clones are closely related to three different bacteria that all come from the coleopteran *Pachnoda epipphiata*, [50] which also hosts the closest relatives to some of the Bacteroidetes clones (Table 2). Also, closest sequences to the clones affiliated with the Clostridiales and some Proteobacteria have been retrieved from the gut of the *Melolontha melolontha* beetle, while several Clostridiaceae clones and one Bacteroidetes clone were closely related to sequences that were all retrieved from the same dipteran host *Tipula abdominalis* gut (GU451010). Given the low homologies and the recurring multiple instances it appears highly unlikely that these occurrences could be coincidental, constituting a significant element in favour of distant but conserved host-bacteria interactive relationships, in which given subsets of bacterial taxa seem to co-occur in a number of parallel situations hosted by very different insects.

**Figure 6:**
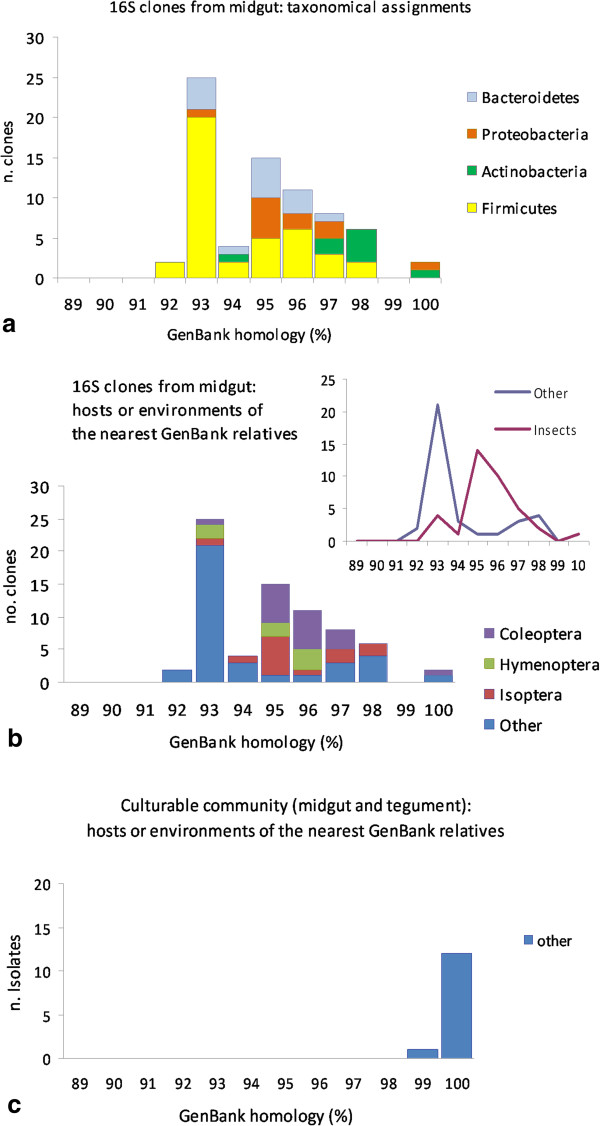
**Phylotype and host partitioning in GenBank subjects with similarity to Cansiliella-associated bacteria. a**) Abundance of 16S rDNA phylotypes found from the midgut using a culture-independent approach and respective GenBank homology percentage classes. **b**) Proportions of insects orders or other environments hosting bacterial subjects resulting in different degrees of sequence homology (x axis) with clones of the non-culturable microbial community from the midgut. The smaller diagram in the upper right corner shows the same data as line graphs and by pooling the insect orders together to put in evidence the separation from the cases found in non-insect environments. **c**) Proportions of insects orders or other environments hosting bacterial subjects resulting in different degrees of sequence homology (x axis) with culturable microbial community isolates from the midgut and external tegument. The definition ‘other’ includes all non-insect guts, faeces, and other habitats as reported in Table 2.

In order to better visualize the distribution of bacterial phyla found in *C. servadeii* along with that of the hosts/habitats where their closest GenBank relatives had been found, in Figure 6 we plotted these across the span of 16S homology at which the BLAST match was found for each clone or isolate. Interestingly, for the midgut clones, the identity levels show a bimodal distribution. Figure 6a shows the distribution of the bacterial taxonomical divisions found within *Cansiliella*’s gut assemblages. When the same are inspected as regards the habitat of the nearest database subject (Figure 6b), a distinction arises separating the insect-related cases (higher homology region, peaking at 95%) from the rest of non-insect environments including mammal guts/faeces, etc., (more distant homology region peaking at 93%). The two peaks (93% and 95%) are significantly different (Wilcoxon Mann–Whitney test, *p*<0.01) (Figure 6b). The fraction of culturable bacteria instead (Figure 6c) displays high levels of similarity shared in all cases with non-insect GenBank subjects.

## Discussion

*Cansiliella* spp. mouthparts are distinct from other cave beetles, in general and from the large majority of the Leptodirini, and show features uncommon to beetles with more saprophagous diets [28]. The beetles in Grotta della Foos have also a semi-aquatic lifestyle associated with moonmilk, which is a rich microbiological substrate mixed with carbonate minerals. Our previous stable isotope investigations, and observations of moonmilk particles in beetle mouths, reveal that *C. servadeii* from Grotta della Foos derives nutrition from moonmilk and habitat waters which contain dissolved organic carbon at a concentration of 10.11 mg/l [30]. The present data show that the insect midgut hosts a bacterial community whose members, as far as it can be judged from the sequenced clones, appear to belong to heterotrophic guilds. The midgut of the insect contains live bacterial cells whose culture-independent analysis yielded a bacterial assemblage dominated by the phyla Firmicutes and featuring presences of Bacteoridetes, Actinobacteria, together with Alpha-, Beta- and Deltaproteobacteria. A possible role of these bacteria in nutritional physiology with activities within the nitrogen metabolism could be postulated on the basis of parallel examples in other gut systems.

The sampling depth proved suitable as this community structure was already fully outlined in terms of phyla and their proportions from the first round of 46 clones. Upon nearly doubling the number, the whole set of 87 clones maintained the same pattern as the new sequences merged into groups which had already appeared. (Additional file 1: Material S1 and Additional file 2: Material S2 vs. Figure 4 and Figure 5).

Interestingly, as seen from each of the subject score lists of the BLAST analysis, the identities of the *C. servadeii* gut bacteria did not overlap with any of the sequences already obtained from our parallel project targeting the bacteria in the moonmilk of the very same cave [39]. In that work, 169 sequences are described (and are available in GenBank under the accession numbers from EU431666 to EU431834). Although moonmilk biota encompassed phyla belonging to the Bacteriodetes, Firmicutes, and *Betaproteobacteria*, there was no OTU overlap (no BLAST identity nor close similarity) between the potentially ingested moonmilk bacteria and the gut-hosted community described in the present report.

These findings confirm the presence of a gut microbiota specificity in *C. servadeii* similarly to what is found in the gut of some insects such as soil or humus-feeding termites [51], european cockchafer larvae (*Melolontha melolontha*) [52] and scarab beetle larvae (*Pachnoda* spp.) [50,53]. For these insects no correspondence has been found either between the gut community and the microbiota of their soil-related diet. On the contrary in insects having a more diverse and richer diet such as crickets and cockroaches higher correspondence between diet and gut bacterial flora has been identified in culture-dependent studies [54,55].

While the uncultured clone library community had such far divergence from known database entries, the culturable bacteria isolated from external tegument and midgut showed a much higher sequence similarity to previously retrieved sequences available in GenBank. Approximately 86% of these sequences have close or equal to 100% sequence similarity (average 97%) (Table 1). In contrast, the uncultured gut clone sequences have lower homology to any previously described bacterial species or environmental sequences, with some as low as 92% (Table 2, Figure 6). Among the dominant OTUs groups, belonging mostly to Firmicutes and Bacteriodetes phyla, sequence similarity with described taxa is ~92% and 94%, respectively, which suggests novel bacterial lineages at the genus-level, if not higher taxonomic ranks. Such result is nowadays an unusual occurrence as the GenBank database contains a large, ever-expanding number of sequences obtained from many different microbiological environments, and it is therefore no longer common to find such low sequence homology, especially when working with a set of several different sequences, all of which turned out consistently distant from known records. Except for two clones corresponding to OTU 14 and OTU 16 that show 100% identity with the Actinobacteria *Sanguibacter inulinus* isolated from the gut of *Thorectes lusitanicus* (Coleoptera Geotrupidae) and *Brevundimonas* sp. isolated from the soil, the rest of the bacterial communities isolated from the gut of *C. servadeii* are highly different from bacteria typical of other gut systems studied until now by culture-independent methods.

Noteworthy, for a number of different groups of taxonomically distinct bacteria hosted by the cave beetle, the insect hosting the closest relatives of each case turned out to be the same (Table 2). For example, the sequences of given firmicutes, bacteroidetes and betaproteobacteria happen to have their top matching GenBank subjects all occurring within the *Melolontha* scarab. Others, also encompassing different phyla have their relatives coinciding within a coleopteran of the *Pachnoda* genus, other clusters co-occur in the Dipteran *Tipula abdominalis*, others within the termite *Reticulitermes speratus*. Given the peculiarity of the sequences, these repeated occurrences appear non-coincidental and support the hypothesis of a selection ensuring the maintenance of a given microbial assemblage for a relevant physiological scope.

Because of the semi-aquatic feeding behaviour of *C. servadeii*, it has been speculated that its ancestor, like that of other hygropetric coleopterans, may have been aquatic [32]. Neverthelesss, considering that the *C. servadeii* gut microbiota having the highest degrees of homology (95-98%) to previously retrieved sequences from invertebrate gut bacteria that spend at least a part of their biological cycle in the soil (Table 2, Figure 4), and mainly to insects belonging to the Isoptera and Coleoptera orders, one could in alternative speculate that the *C. servadeii* ancestor had a terrestrial origin. However in available databases, bacteria from aquatic insects could be still poorly represented to enable a thorough assessment in this regard. About these aspects, a survey of microbial phylotypes from the guts of the other species in the genus, and a barcoding comparison of the insect genes are envisaged as parts of future research.

Considering the evolutionary history of the *C. servadeii* and its gut symbiont system, a long history of separation from other invertebrates and microorganisms appears to have occurred. At the same time its situation reveals the existence of phylogenetic similarities across the digestive tracts of many different hosts (Table 2). It is conceivable that there may be a common ancestry involving a functional guild of bacteria that has endured the host lineage separation, as well as the erosion of sequence identities, through the paths of independent evolution. The dual pattern of homology among clone sequences from gut bacteria in *Cansiliella* to other insects further suggests this scenario (Figure 6b); a progressive phenomenon of divergence from common ancestries is suggested by the double-peaking instance of homology existing between *C. servadeii*’s sequence queries and GenBank subjects, that set the insect-dwelling cases separated from the general intestinal/faecal cases. It is noteworthy that, while the hosts are set apart by sequence homology thresholds, the taxonomical groups of the bacteria found in *Cansiliella* are rather evenly represented across the different homology span (Figure 6a). It can be seen that Firmicutes, Bacteroidetes and Proteobacteria are almost equally present throughout the sequence similarity gradient, underscoring the need of the whole functional assemblage to be conserved both in distantly- as well as in recently-diverged hosts. This emphasizes a supposedly crucial role of a well-defined set of prokaryotic taxa that appear to have remained in charge within the alimentary tract of animals in spite of ages of separation of their hosts.

More recent acquisitions across different hosts appear to correspond to higher degrees of homology for bacterial symbionts, while acquisitions and symbiotic associations that are older would correspond to lower degrees of homology (Figure 6). The evidences depicted in Figure 6 appear to fit the contour of an evolutionary path of separation of the midgut bacteria from those of other insects; it appears that matching bacteria that are hosted in other insects (i.e. hosts that are closer to *Cansiliella)* share higher homology with its symbionts (peak at 95%), while those living in animals which are evolutionarily more distant from the beetle, or in other habitats, have undergone a correspondingly higher divergence from them (peak at 93%). These instances support the existence of a group of common ancestors for a set of different bacteria and a history of isolation and coevolution within the hosts. The same analysis performed with the culturable biota isolated from the external tegument or, as a minority, from the midgut, shows the opposite scenario (Figure 6c) i.e. a high level of similarity with non-insect environments (Table 1), suggesting that plate-culturable taxa are also more prone to spread/reproduce and be part of a more diffuse cosmopolitanism.

## Conclusions

The insects hereby examined feature a defined gut community of bacteria suggesting a long history of inheritance and a coevolution.with their hosts. Corresponding, but genetically diverged, microbial assortments appear to exist, in parallel, in a series of other animals’ digestive systems. It appears that the reproductive boundaries arisen between the hosts at their speciation stages, have, at the same pace, prevented the exchange of their gut bacteria. The conservation of these sets of prokaryotic taxa suggests a relevant role in animal physiology.

The evidence of such patterns casts light on their biology at both physiological and evolutionary scales. Elucidating, in future studies, the details of the bacterial transmission in *C. servadeii* will offer useful insights to further interpret bacterial evolution and the critical roles of prokaryotes in the animal-microbe interactions ecology.

## Competing interests

The authors declare that they have no competing interests.

## Authors’ contributions

MGP defined the whole experimental plan of the research, organized the fieldwork and identified the zoological samples; LM, MS and IMS performed the gut microscopy and the cloning and sequencing of microbial 16S genes and constructed the phylogeny trees; ALD, AP, MB and LD organized the logistics of the speleological expedition into the cave, collected the insect samples and recorded their in-situ behaviour, ASE provided the data of microbial colonization of the cave substrate moonmilk and discussed its similarity with the *Cansiliella* microbiota; AT and BB performed the fluorescent stereomicroscopy detection of bacteria on external appendages of the insect; GC performed the water chemical analysis of the cave environment; AS performed the bioinformatical analyses, the microbial ecology assessment and wrote the manuscript. All authors read and approved the final manuscript.

## Supplementary Material

Additional file 1 Cluster analysis dendrogram obtained with the first 46 screened clones, Gram-negative portion. Click here for file

Additional file 2 Cluster analysis dendrogram obtained with the first 46 screened clones, Gram-positive portion. Click here for file

Additional file 3 Rarefaction curve for OTUs defined at 81% similarity. Click here for file
