# Supplementary figures and images for: A unique midgut-associated bacterial community hosted by the cave beetle Cansiliella servadeii (Coleoptera: Leptodirini) reveals parallel phylogenetic divergences from universal gut-specific ancestors
Source: BMC Microbiol. 2013 Jun 10;13:129. doi: 10.1186/1471-2180-13-129 (PMC3695770; doi:10.1186/1471-2180-13-129)

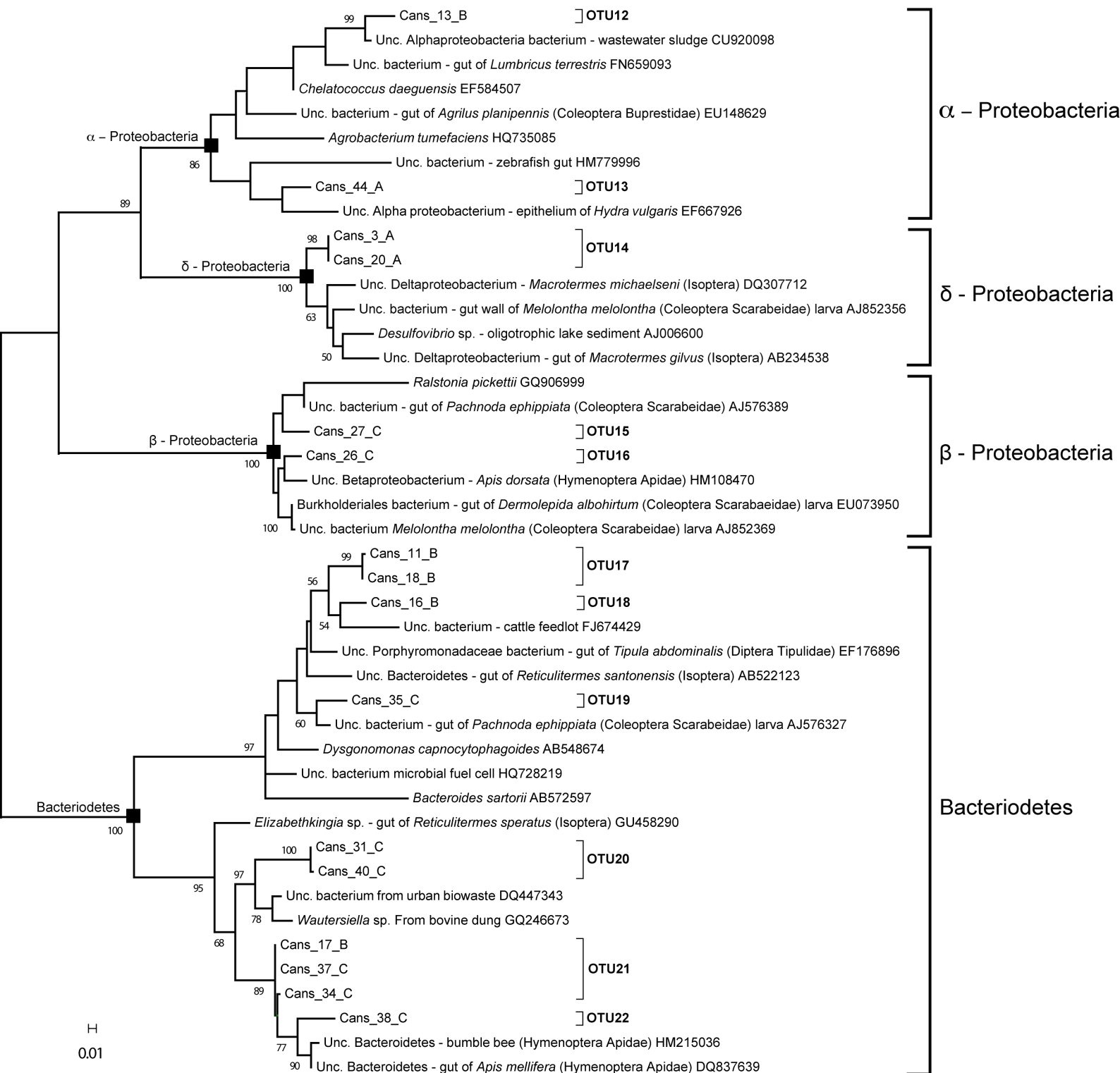

Supplement: Additional file 1 — Cluster analysis dendrogram obtained with the first 46 screened clones, Gram-negative portion. [file 1471-2180-13-129-S1.pdf]

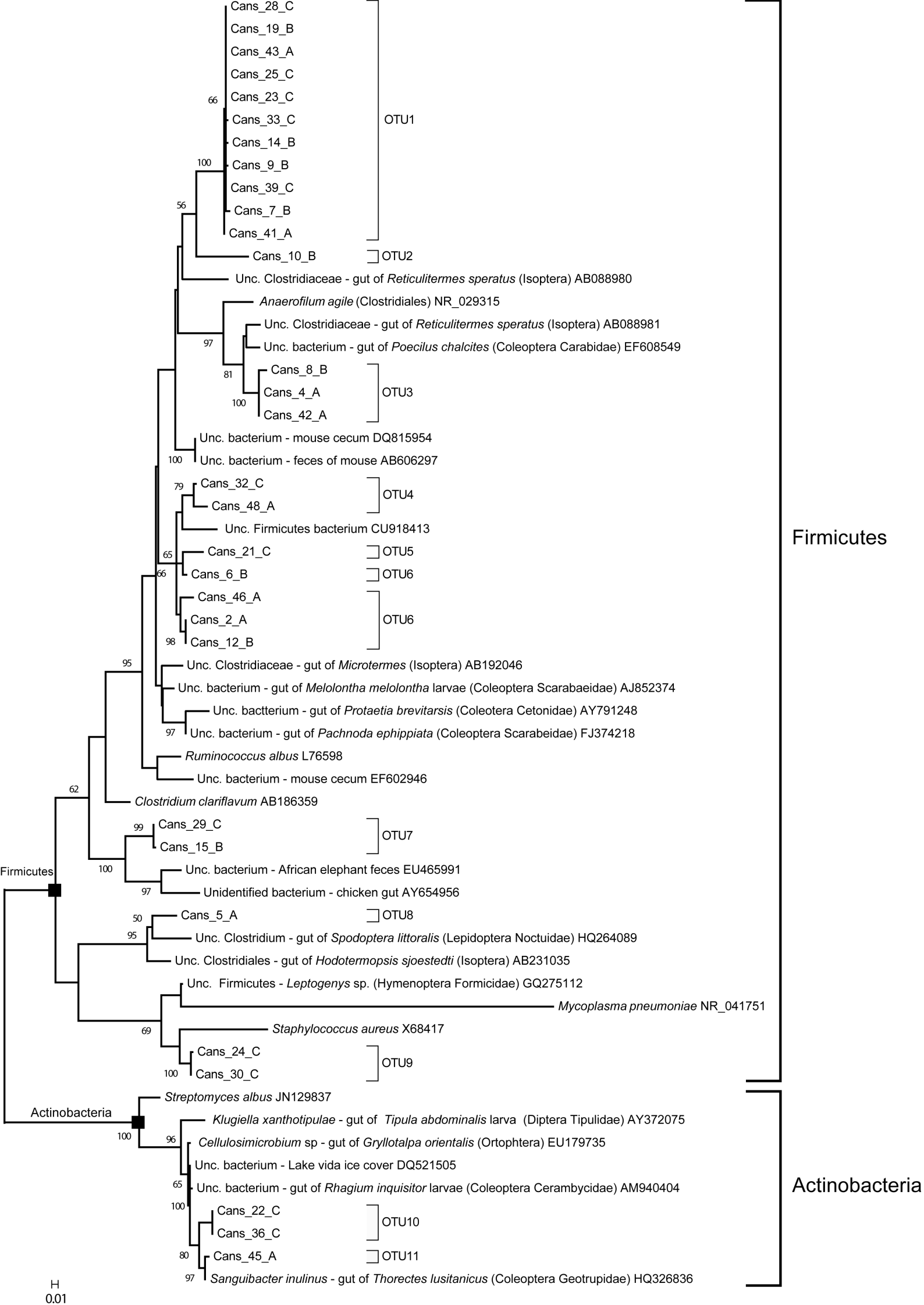

Supplement: Additional file 2 — Cluster analysis dendrogram obtained with the first 46 screened clones, Gram-positive portion. [file 1471-2180-13-129-S2.pdf]

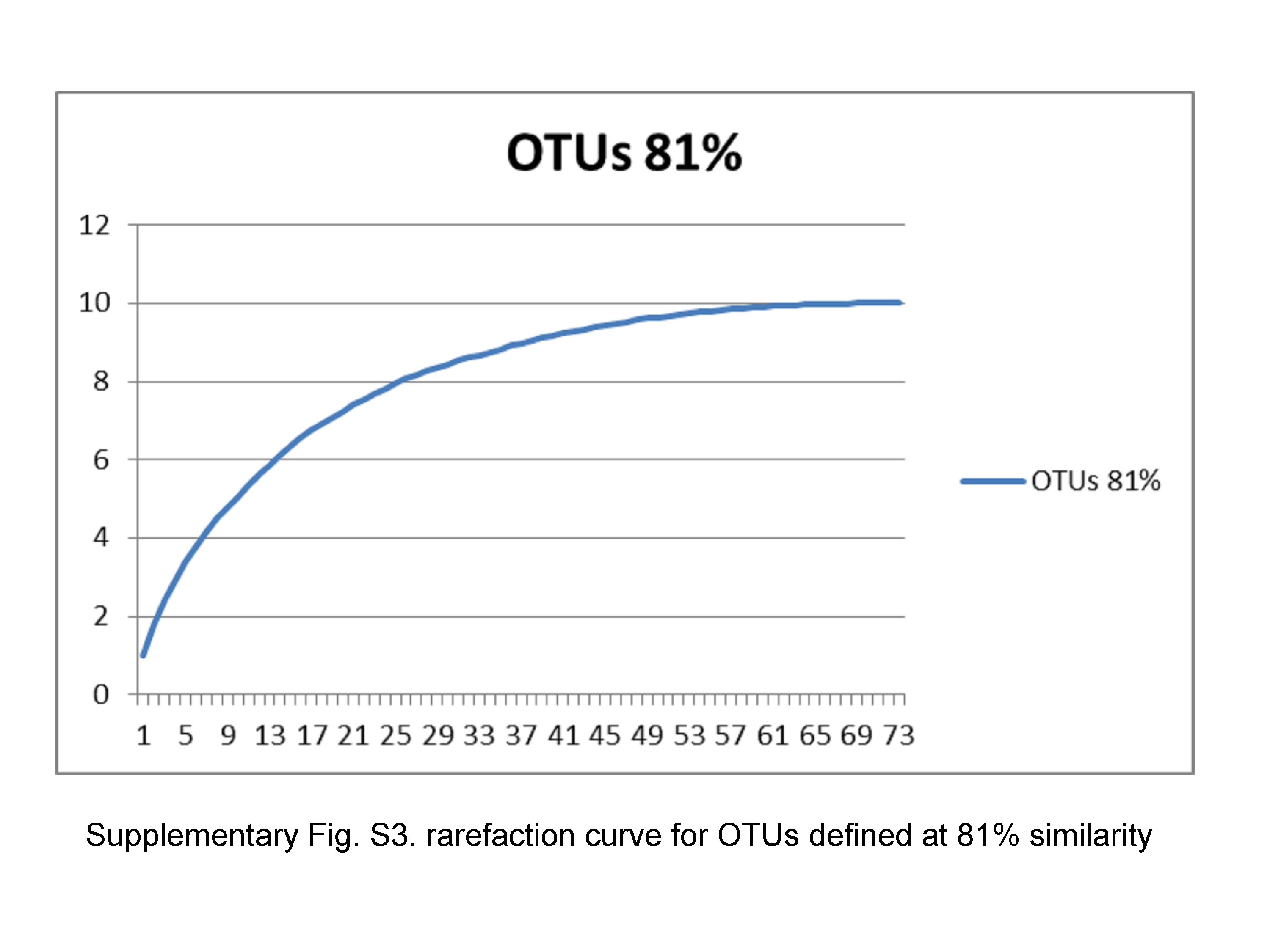

Supplement: Additional file 3 — Rarefaction curve for OTUs defined at 81% similarity. [file 1471-2180-13-129-S3.tiff]
